# Transforming psychological practice with precision mental health: introduction to the NOVA project

**DOI:** 10.3389/fpsyg.2026.1775489

**Published:** 2026-02-27

**Authors:** Pablo Roca, Sara Noheda, Eduar Ramírez-Riveros, Carolina Martín-Azañedo, Fermín Martínez-Zaragoza, Rosaria Maria Zangri, Eduardo P. García del Valle, Martin Sanchez-Pedreño, Montserrat León-Garcia, Derek Loy Gravholt, Angel Enrique, Rob Saunders, Luis Javier Herrera, María del Carmen Pegalajar, Carmelo Vázquez, Sara Rodriguez-Moreno

**Affiliations:** 1 Precision Mind Lab (Premind), Universidad Villanueva, Madrid, Spain; 2 Medea Lab, Madrid, Spain; 3 Universidad Francisco de Vitoria, Pozuelo de Alarcón, Spain; 4 Department of Behavioural Sciences and Health, Miguel Hernandez University of Elche (UMH), Alicante, Spain; 5 Facultad de Salud, UNIE Universidad, Madrid, Spain; 6 School of Science and Technology, IE University, Madrid, Spain; 7 Knowledge and Evaluation (KER) Unit, Mayo Clinic, Rochester, MN, United States; 8 Amwell Science, Amwell, Boston, MA, United States; 9 CORE Data Lab, Research Department of Clinical, Educational and Health Psychology, University College London, London, United Kingdom; 10 Department of Computer Engineering, Automatics and Robotics, University of Granada, Granada, Spain; 11 Department of Computer Science and Artificial Intelligence, University of Granada, Granada, Spain; 12 Complutense University of Madrid, Madrid, Spain; 13 Faculty of Biomedical and Health Sciences, Universidad Europea de Madrid, Madrid, Spain

**Keywords:** artificial intelligence, clinical decision support systems, data-informed decision making, implementation science, measurement-based care, precision mental health

## Abstract

Evidence-Based Practice (EBP) has increased the availability of psychological treatments, yet many people do not benefit from therapy, some report deteriorations in symptoms, and dropout rates remain high. Precision Mental Health (PMH) is proposed as an extension of EBP by combining systematic measurement with predictive analytics to support the right intervention, at the right time, for the right person. Recent advances in Artificial Intelligence (AI) make PMH increasingly feasible in routine psychotherapy; however, the implementation of these approaches in routine care is still incipient. In this context, the present article has two main aims. First, we summarize key advances in PMH, particularly measurement-based care and data-informed decision making. Second, we introduce the NOVA project (Navigating Outcomes via Analytics), a multi-phase translational program designed to implement PMH in real-world psychological services. Guided by the Implementing Precision Methods framework, NOVA integrates (i) stakeholder-informed work on clinician acceptability and intention to use, (ii) pragmatic evaluation of decision support tools in routine care, (iii) development of robust and interpretable predictive models, and (iv) training and dissemination activities aligned with responsible innovation and professional competencies for AI-supported precision care. By detailing NOVA’s implementation pathway, we aim to provide a concrete roadmap for bridging AI innovation and psychological practice, accelerating the sustainable adoption of PMH in real-world settings.

## Introduction

Over the last three decades, Evidence-Based Practice (EBP) has been the dominant paradigm in mental healthcare. Randomized controlled trials (RCTs), meta-analyses and clinical guidelines have provided a robust answer to the question: “On average, does this treatment work better than no treatment or an alternative?.” Multiple treatments (e.g., cognitive-behavioral therapy, mindfulness, pharmacotherapy…) show moderate average effects in the treatment of depression and anxiety disorders, leading to guidelines recommending these as first-line interventions (e.g., National Institute for Health and Care Excellence, 2022; American Psychological Association, 2019; World Health Organization, 2019). However, classic EBP has three well-documented limitations in informing clinical practice:

- It is built on group averages, but we treat individuals: unsurprisingly, psychological treatments designed for the average patient often led to average results, and interventions that demonstrate efficacy for one individual may be less effective or iatrogenic for another, thereby underscoring the limitations of the ‘one-size-fits-all’ approach (Purgato et al., 2021). Evidence suggests that nearly half of patients undergoing psychological therapy fail to achieve clinically meaningful improvements and approximately 10% experience worsening symptoms (Schwartz et al., 2021).
- Treatment selection often relies on ‘trial and error’: although many outcome predictors have been identified, this knowledge has not yet translated into real-world treatment recommendations guiding which therapy to choose for whom (DeRubeis et al., 2014). Therefore, therapy non-completion (i.e., unilateral termination of therapy by the patient) remains a significant issue, with dropout rates estimated at around 30–40% (Cooper and Conklin, 2015). Importantly, a proportion of dropout occurs at very early stages of care (often within the first one to two sessions), before a specific treatment approach could reasonably be implemented, which may reflect factors such as expectations, logistics, or early therapeutic fit rather than ‘trial-and-error’ with treatment techniques per se (Fernandez et al., 2021).
- Complex real-world presentations exceed guideline simplicity: predictors of outcome such as contextual factors, comorbidity, chronicity, treatment history, service constraints… interact with treatment characteristics and therapist factors in complex ways that are rarely captured by group analyses.

In practice, this means that applying “the treatment of choice for diagnosis X” often leaves a significant proportion of patients insufficiently helped and often under-specify how to personalize the intervention or what to do next when a patient is not improving. Surprisingly, despite improved and more widely disseminated psychological treatments, rates of depressive and anxiety disorders in the general population have not fallen, which have been described as the “treatment–prevalence paradox” (Ormel et al., 2022). Taken together, these findings suggest that the traditional EBP model is insufficient and the lack of personalization remains a critical factor, undermining the effectiveness of prevention, diagnosis, and treatment efforts.

Personalization has always been part of psychological practice, but it has been relatively unsystematic and guided primarily by clinical judgment. However, there is growing evidence showing that clinical judgment alone is limited for complex decisions such as when to change course in treatment, how to match a patient to a therapist, or which intervention components to prioritize. Classic work comparing clinical versus statistical prediction indicates that mechanical or actuarial methods of combining data are about 10% more accurate than unaided clinical judgment with experience-based predictions made without explicit actuarial rules, statistical models, or algorithmic decision support (Grove et al., 2000). In psychotherapy, studies repeatedly find that therapists substantially underestimate the proportion of individuals who will deteriorate and overestimate the likelihood of improvement (Østergård et al., 2024). Moreover, large naturalistic datasets suggest that therapists’ average outcomes do not improve over years of experience (Goldberg et al., 2016), challenging the assumption that expertise alone corrects this bias. Even well-established EBPs are often under-implemented or delivered with substantial variability across routine care settings, reinforcing the science-to-practice gap and limiting population-level impact (Herschell et al., 2010).

Given the limitations of traditional EBP and the clinical judgment, a family of “precision-based approaches” has emerged as a natural extension, guiding more tailored and data-informed decisions for each individual patient. The concept of “precision” comes originally from general medicine. Precision Medicine aims to tailor prevention and treatment to the characteristics of each individual, using information on genes, environment and lifestyle to move beyond one-size-fits-all protocols (Collins and Varmus, 2015). In mental health, this idea has evolved into Precision Mental Health (PMH; also known as Precision Psychiatry or Precision Psychology), an approach to prevention and intervention that seeks a more accurate understanding of each person’s needs, context, preferences, risk profile, and likely prognosis, in order to guide more precise decisions about assessment, treatment selection, monitoring and adaptation (Bickman et al., 2016). PMH aims to deliver the right intervention, at the right time, by the right clinician, for a given individual, using data to inform these choices rather than relying solely on averages or intuition (Delgadillo and Lutz, 2026), in press). This is not a rejection of EBP, but an extension of it: it aims to determine which evidence-based option, in what sequence or combination, is likely to be optimal for this particular person, and how we can update that plan as new data arrives. Because EBP is always delivered in the context of a real encounter (i.e., a conversation between clinician and patient), the evidence must be translated and personalized in that dialog (León-García et al., 2023).

## What does precision mental health look like in clinical practice?

**Figure 1:**
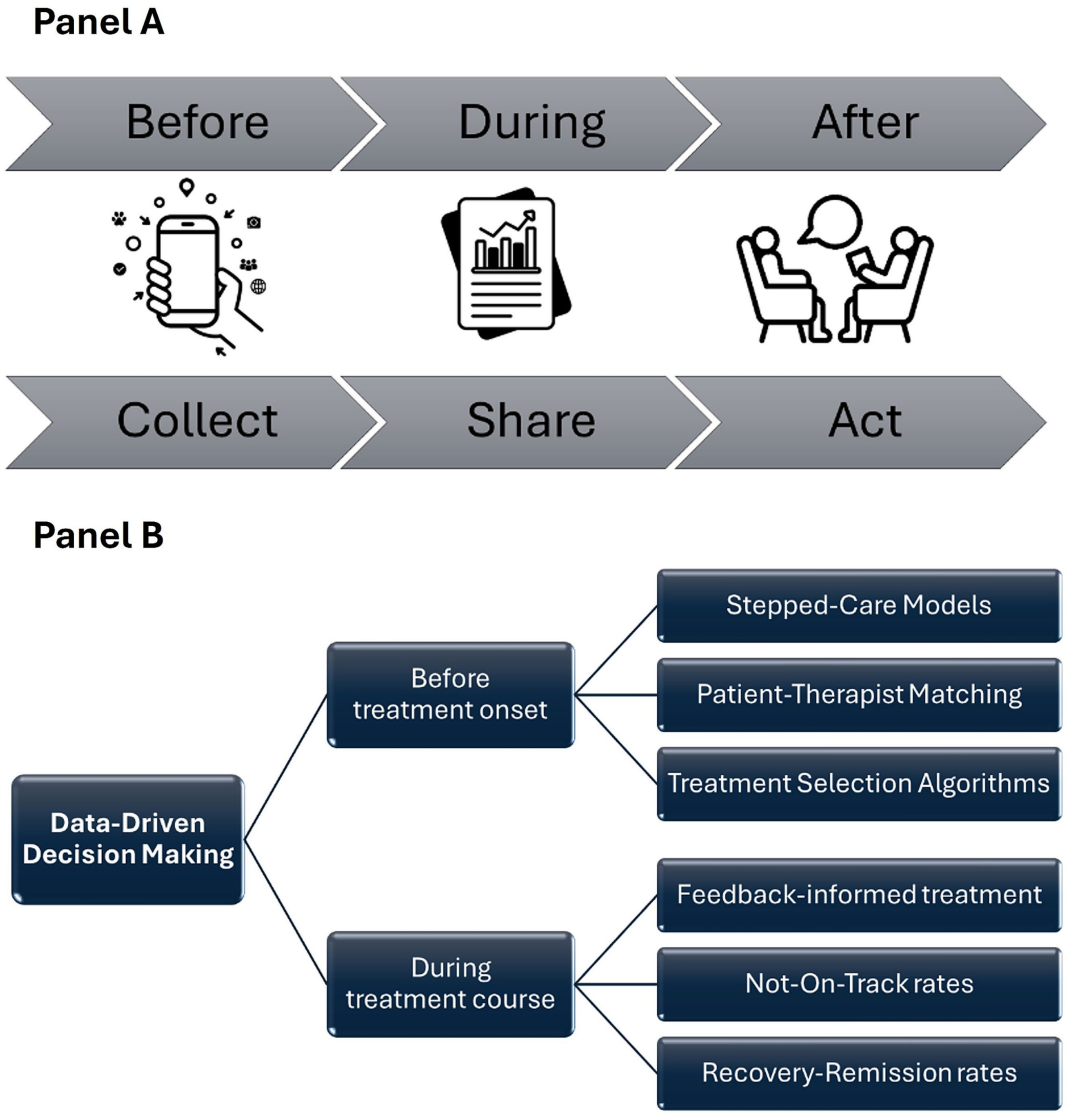
Precision mental health main pillars: **(A)** Measurement-based care, **(B)** Data-driven decision making.

Conceptually, the field has evolved along a development pathway: from identifying prognostic factors, to building and validating prediction models, and ultimately to implementing decision support tools that can guide treatment selection and ongoing adaptation in routine care (Delgadillo and Lutz, 2020). Within this pathway, conceptual and empirical work converges on two main pillars of PMH services (Bickman et al., 2016; Lutz et al., 2022a; Lutz et al., 2024):

- Measurement-Based Care (MBC): in PMH assessment is not an optional add-on but the infrastructure that makes prediction, personalization and adaptive intervention possible. MBC is the systematic assessment of clinically relevant information (e.g., outcomes, processes, preferences, context, etc.), becoming the backbone of data-informed decision-making. MBC integrates three core features (Barber and Resnick, 2023): (1) Collect: collecting patient data on regular basis; (2) Share: providing visual feedback on psychotherapy progress; and (3) Act: adapting the focus or direction of therapy based on the feedback received (see Figure 1A). Compared to traditional practice, where progress is judged primarily by clinical judgment, MBC enables timely adjustment of treatment intensity or format by making change visible early. It also strengthens shared decision-making, as progress feedback (e.g., brief progress graphs or risk alerts) can be discussed collaboratively with patients to refine goals and expectations and to guide adaptations in the treatment plan. Finally, when aggregated across patients and clinicians, MBC data creates a naturalistic learning system that can inform service-level quality improvement and the refinement of prognostic tools and personalization strategies over time. In addition to clinical benefits, aggregated MBC data can support organizational decision-making by demonstrating service impact, informing program leadership and policy discussions, and strengthening the justification for implementation resources (e.g., training time, technology procurement) or continued funding.
- Data-Driven Decision Making (DDDM): continuous assessment generates valuable data, but data alone does not guarantee better decisions. Therefore, the second building block of PMH is the use of predictive models to transform raw measurements into clinically usable recommendations. DDDM refers to the structured use of systematically collected data combined with algorithms and statistical models to inform decisions about care while keeping psychologists accountable for final judgment and action (Tanguay-Sela et al., 2022). As mentioned above, a substantial body of research has consistently demonstrated that statistical models frequently outperform clinical judgments, particularly among less experienced practitioners. Ranging from relatively simple prognostic models to more complex Artificial Intelligence (AI) and Machine Learning (ML) algorithms, predictions can be used to estimate prognostic models on likely outcome trajectories, to inform treatment selection, treatment intensity or modality in stepped-care models, therapist–patient matching, or to trigger timely feedback or clinical risk alerts (see Figure 1B). This rationale closely aligns with large-scale, data-informed care pathways such as the IAPT/NHS Talking Therapies program (Wakefield et al., 2021), where routine outcome monitoring and algorithmic decision support are increasingly used to guide stepped-care decisions.

**Figure 2:**
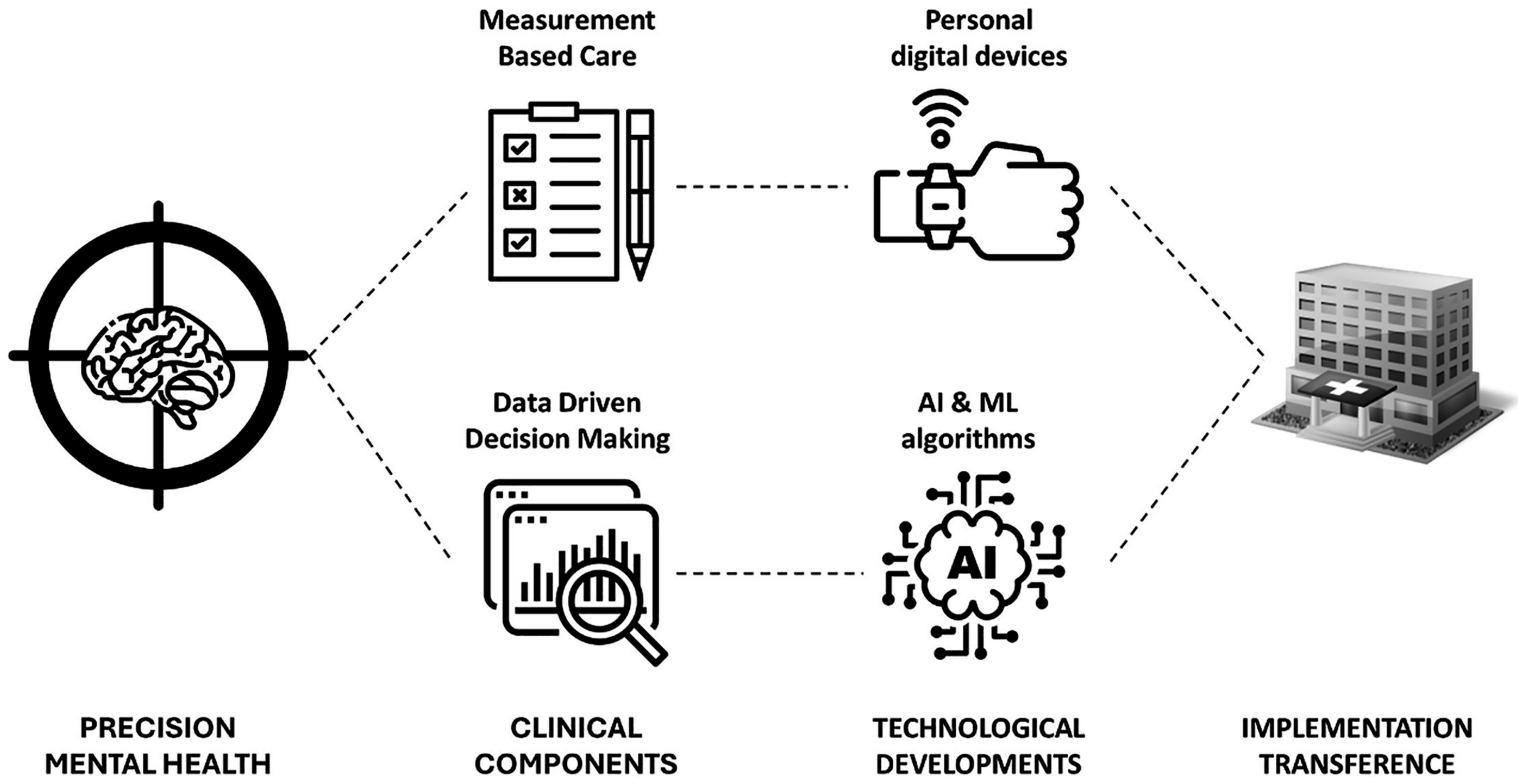
Precision mental health conceptual framework.

Over the past decade, mental health care has undergone a technological revolution, accelerating the digitization of psychotherapy (Domhardt et al., 2021), a trend further intensified by the COVID-19 pandemic (Appleton et al., 2021). Two complementary technological innovations have facilitated the implementation of PMH into clinical practice (Figure 2). First, Personal Digital Devices such as smartphones, wearables, and other sophisticated mobile technologies have significantly streamlined Experience Sampling Methods (ESM) and Ecological Momentary Assessment (EMA), enabling real-time, within-subject ambulatory assessments that capture intensive longitudinal data (Aan het Rot et al., 2012). These devices also support Digital Phenotyping (Montag et al., 2020), which uses sensor and usage data to infer contextual and behavioral patterns that can be leveraged to predict mental health status. Second, the vast databases generated through these assessment technologies can be analyzed using advanced AI and ML algorithms (Rutledge et al., 2019). Several studies have highlighted the potential advantages of applying AI and ML in mental health (Roca, 2025), for example, predicting the risk of mental health conditions, case formulation, treatment selection or reducing administrative and documentation burden.

## PMH implementation challenges

Given the growing body of scientific literature demonstrating the positive impact of PMH technologies on treatment outcomes and adherence (Nye et al., 2023), both the American Psychological Association and the Roadmap for Mental Health Research in Europe have advocated for the integration of PMH technologies into routine clinical practice. However, implementation in clinical practice has been limited, resulting in a gap between scientific advancements and mental healthcare delivered in real-world settings. Implementation studies highlight numerous barriers that must be addressed for PMH to move from proof-of-concept models to standard practice. For instance, estimates suggest that only 14% of clinicians used progress measures (Jensen-Doss et al., 2018), and fewer than 20% employed PMH methods (Lewis et al., 2019). Moreover, a recent systematic review revealed that fewer than 1% of individualized prediction models in mental health research have been evaluated for potential implementation in real-world clinical settings (Salazar de Pablo et al., 2021).

Adopting PMH is not simply a matter of “plugging in” an algorithm, it entails cultivating new competencies (e.g., data literacy, critical appraisal), engaging with ethical and legal questions (e.g., consent, secondary use of data), and participating in organizational decisions about how these PMH systems are implemented and evaluated. This perspective is articulated in the Implementing Precision Methods (IPM; Deisenhofer et al., 2024) framework, which classifies implementation challenges into four main domains: (1) Attitudinal domain: effective implementation of PMH technologies requires addressing the needs and concerns of both clinicians and clients. Clear clinical value (e.g., fewer dropouts, better outcomes), loss of autonomy, damage to the therapeutic relationship, measurement burden for patients, integration into existing workflows, increased workload… are some of the critical determinants of PMH adoption; (2) Technological domain: Software and hardware challenges such as ensuring secure and efficient technological infrastructure, user-friendly interfaces, interoperability with electronic health records (EHR)… may pose barriers to the implementation of PMH technologies; (3) Statistical domain: When implementing PMH technologies, clinicians must have confidence in key features of the algorithms they rely on. Overfitting, poor generalizability in new populations, algorithmic bias (e.g., systematically under- or over-estimating risk for specific sociodemographic groups), long-term accuracy, transparency vs. black box… are issues that can undermine both accuracy and ethical acceptability; and (4) Contextual domain: Certain challenges may arise in specific settings where service policies, organizational culture, and available resources are not aligned to support the adoption of PMH technologies. Professional training, organizational culture, regulatory frameworks, reimbursement models… shape whether PMH tools are used as intended or sidelined.

There is a rapid development of PMH technologies to help clinicians choose and adapt interventions for individual patients. Clinical decision support systems (CDSS) embedded into EHR or dedicated platforms are one of the most widespread ways of translating PMH into real-world settings (Lutz et al., 2022b). CDSS translate the core ingredients of PMH into therapists’ daily decision-making, presenting predictions and recommended actions in a user-friendly way (visual dashboards, alerts, language models, etc.). Rather than replacing clinicians, CDSS presents predictions and recommendations that therapists can adopt or override, functioning as a form of technology-augmented case formulation. Clinical case studies illustrate how CDSS can enhance therapist awareness of subtle negative trends and support decisions such as adding specific modules, revisiting the therapeutic alliance, or considering referral to higher levels of care (Lutz et al., 2024). Internationally, there are some examples such as the Trier Treatment Navigator (TTN; Lutz et al., 2019), a CDSS developed in a university-based training clinic that integrates routine outcome monitoring with risk algorithms to provide case-level feedback, “off-track” alerts, and data-informed suggestions for case formulation and treatment adaptation. In Spain, platforms such as Psypilot are beginning to translate these ideas into scalable, practice-oriented software solutions for mental health professionals. Unlike other CDSS, Psypilot leverages AI to enhance implementation by offering a virtual assistant that interprets PMH recommendations through large language models acting as a clinical copilot for the professional.

## Discussion

Within this landscape, the NOVA (Navigating Outcomes via Analytics) project was born with the commitment to investigate how to implement PMH in the messy reality of everyday psychological practice. The project focuses on three recurrent challenges in clinical practice: suboptimal outcomes, high dropout and limited use of data in decision-making- and tackles them through an integrated package of attitudinal research, clinical trials, algorithm development and policy translation. The NOVA project was explicitly designed around the two PMH pillars, combining measurement-based care with data-informed decision support, while using the IPM framework to anticipate and address implementation barriers. In doing so, the NOVA project aims to offer a concrete model for how PMH can move from theory and isolated pilots to sustainable use in everyday psychological practice.

NOVA is not limited to adopting new measures, algorithms or digital tools but involves embedding a whole PMH pipeline -from systematic data collection to predictive modeling and decision support- within routine services. Therefore, each work package is designed to address one or more IPM domains: (1) Attitudinal studies map psychologists’ attitudes, perceived benefits and concerns, informing them how PMH technologies should be designed, communicated and translated; (2) Clinical trials of a decision support system examine not only patient outcomes but also usability, workflow fit and impact on therapeutic processes; (3) ML work focuses on robust, interpretable models that can be updated and audited, rather than “black box” prototypes; and (4) Policy and dissemination activities explicitly connect project findings to national strategies in mental and digital health.

NOVA is a multi-phase program structured around sequential phases that move from stakeholder acceptability and implementation determinants, to pragmatic evaluation of a CDSS, to the development and pilot validation of prediction algorithms, and finally to training and policy translation (see Table 1). The project is conducted by a multi-stakeholder consortium combining clinical science, data science, implementation support, and real-world service partners. The project leadership and clinical research expertise are based in the Precision Mind Lab at Universidad Villanueva (Madrid, Spain), and the AI/ML and data mining expertise is provided by the Technical School of Computer and Telecommunication Engineering at the University of Granada. Routine-care implementation is enabled through a network of service partners that include hospital and outpatient clinic settings and broad professional networks. The CDSS platform used in the pragmatic trial is provided by the technological innovation partner Medea Mind, which supports adaptation, integration, and technical assistance for real-world deployment. International advisors contribute complementary expertise in PMH, digital health implementation, and outcomes research (e.g., University College London, Mayo Clinic, Complutense University).

**Table 1:** Overview of NOVA phases, designs, settings, and deliverables.

Phase	Aim	Design	Participants	Outcomes	Deliverables
1	Identify determinants of acceptability and intention to use PMH technologies among psychologists	Online survey with model-based analyses (e.g., SEM)	Training and practicing psychologists	Acceptability, intention to use, perceived barriers/facilitators; predictors of adoption	Publication on an empirical, theory-guided PMH adoption model
2	Evaluate feasibility, acceptability, and impact of CDSS-supported care in routine psychotherapy	Pragmatic, multisite, 2-arm RCT (CDSS vs. TAU).	Adult outpatients and licensed psychologists in routine-care clinics	Clinical outcomes, functioning, dropout status; satisfaction, alliance; usability/acceptability; engagement/adherence.	Publication on the feasibility and outcomes of the pragmatic RCT
3	Develop and validate ML-based patient–therapist matching to improve fit and outcomes	Two-step approach: (1) model training using Phase 2 data; (2) pilot/feasibility RCT (Match vs. CAU)	Routine-care clinic sample for model training; new pilot RCT sample for validation	Dropout/retention, satisfaction, alliance, clinical change, model performance and explainability	Publication on model development and validation
4	Translate findings into implementable training, guidance, and policy recommendations	Development of training materials and implementation guidance	Clinicians, patients, policymakers, professional bodies	Sakeholder feedback on usability/feasibility of guidance	Publication on the training effects and materials and policy briefs/executive summaries

NOVA is expected to deliver a set of tangible outputs and infrastructures that extend beyond the project’s timeframe: (1) a standardized measurement and data infrastructure for PMH in participating services, including validated assessment batteries, digital workflows and interoperable data pipelines; (2) curated datasets suitable for ongoing research on prognostic modeling, treatment personalization and therapist effects, contributing to international efforts to refine PMH methods; (3) working prototypes of CDSS and algorithms, co-designed with clinicians and patients, and ready for further scaling or integration into existing platforms; (4) a trained community of professionals with competencies in PMH, essential ingredients for the emergence of a “precision psychologist” profile in Spain; and (5) Policy recommendations about facilitators and barriers to implementing PMH, mapped onto the IPM framework and directly connected to ongoing national strategies in mental health.

A critical implementation implication concerns workforce development, and NOVA explicitly treats this as a core target rather than a downstream dissemination activity. In many real-world settings, clinicians have limited formal training in predictive modeling, algorithm evaluation, and uncertainty interpretation, which can foster both under-trust (dismissal of potentially useful feedback) and over-trust (automation bias). Responsible PMH implementation therefore requires explicit competency-building (measurement literacy, basic data interpretation, understanding model limits and uncertainty, and ethical/legal literacy around consent and secondary data use), as well as organizational supports (workflow integration, local champions, consultation access, and supervision norms for discussing when and why to follow or override recommendations). There are emerging examples of integrating routine outcome monitoring and decision support into training and supervision contexts (e.g., university-based training clinics using CDSS such as the Trier Treatment Navigator). Building on these precedents, NOVA’s dissemination and training deliverables are intended to support not only service-level implementation but also integration into supervision structures and training curricula (e.g., guidance materials, workshops/webinars, and practical recommendations for interpreting and discussing PMH feedback in supervision).

## Data Availability

The original contributions presented in the study are included in the article/supplementary material, further inquiries can be directed to the corresponding author.
